# Exploring New Biological Functions of Amyloids: Bacteria Cell Agglutination Mediated by Host Protein Aggregation

**DOI:** 10.1371/journal.ppat.1003005

**Published:** 2012-11-01

**Authors:** Marc Torrent, David Pulido, M. Victòria Nogués, Ester Boix

**Affiliations:** 1 Department of Biochemistry and Molecular Biology, Biosciences Faculty, Universitat Autònoma de Barcelona, Cerdanyola del Vallès, Spain; 2 Department of Experimental and Health Sciences, Universitat Pompeu Fabra, Barcelona Biomedical Research Park, Barcelona, Spain; Northwestern University Feinberg School of Medicine, United States of America

## Abstract

Antimicrobial proteins and peptides (AMPs) are important effectors of the innate immune system that play a vital role in the prevention of infections. Recent advances have highlighted the similarity between AMPs and amyloid proteins. Using the Eosinophil Cationic Protein as a model, we have rationalized the structure-activity relationships between amyloid aggregation and antimicrobial activity. Our results show how protein aggregation can induce bacteria agglutination and cell death. Using confocal and total internal reflection fluorescence microscopy we have tracked the formation *in situ* of protein amyloid-like aggregates at the bacteria surface and on membrane models. In both cases, fibrillar aggregates able to bind to amyloid diagnostic dyes were detected. Additionally, a single point mutation (Ile13 to Ala) can suppress the protein amyloid behavior, abolishing the agglutinating activity and impairing the antimicrobial action. The mutant is also defective in triggering both leakage and lipid vesicle aggregation. We conclude that ECP aggregation at the bacterial surface is essential for its cytotoxicity. Hence, we propose here a new prospective biological function for amyloid-like aggregates with potential biological relevance.

## Author Summary

Microbial infections are reported among the worst human diseases and cause millions of deaths per year over the world. Antibiotics are used to treat infections and have saved more lives than any other drug in human history. However, due to extended use, many strains are becoming refractive to common antibiotics. In this light, new promising compounds, like antimicrobial proteins and peptides (AMPs) are being investigated. Some AMPs also show agglutinating activity; this is the ability to clump bacteria after treatment. This feature is particularly appealing because agglutinating peptides could be used to keep bacteria to the infection focus, helping microbe clearance by host immune cells. In this study, we propose a novel mechanism to explain agglutinating activity at a molecular level using Eosinophil Cationic Protein. We show that the agglutinating mechanism is driven by the protein amyloid-like aggregation at the bacteria cell surface. Accordingly, elimination of the amyloid behavior abolishes both the agglutinating and the antimicrobial activities. This study provides a new concept on how Nature could exploit amyloid-like aggregates to fight bacterial infections. Moreover, these results could also add new insights in understanding the relation between infection and inflammation with dementia and amyloid-related diseases like Alzheimer.

## Introduction

Antimicrobial proteins and peptides (AMPs) represent a wide family that contributes to the host defense system with multiple pathogen killing strategies [1]–[3]. Their fast and multitarget mechanism of action reduces the emergence of bacteria resistance and represents a valuable alternative for common antibiotics [4], [5].

The mechanism of action of AMPs has been systematically investigated, suggesting that AMPs bind to bacteria cell membranes and disrupt cell homeostasis. However, more investigations are needed to completely understand how different structures determine the function of AMPs [6]–[12]. Membrane damage is a multifaceted mechanism that can involve different peptide assemblies and ultimately promotes membrane permeabilization when achieving a critical concentration [13], [14]. Several authors have highlighted the striking resemblance of membrane disrupting mechanisms with those observed for amyloid peptides and proteins [15]–[17]. In both cases, membrane composition (e.g. cholesterol content) and biophysical properties (e.g. membrane fluidity and curvature) were found critical for the peptide action [13], [15], [18]–[26]. Furthermore, we have recently suggested that antimicrobial activity could have arisen through cationization of amyloid-prone regions [27]. In this light, some AMPs have been described to form amyloid structures *in vitro* [28], [29] and some amyloid peptides have also been considered as putative AMPs [30], [31]. In fact, we have proposed that inherent AMP aggregation properties can modulate antimicrobial activity [32].

Interestingly, some antimicrobial proteins and peptides have been found to agglutinate bacteria cells. In this sense, bacteria agglutination has been ascribed to unspecific adhesion through hydrophobic interactions, as observed for synthetic peptides derived from the parotid secretory protein [33]. Comparative analysis on those peptides highlighted the contributions of both hydrophobic and cationic residues in the agglutination activity [33]. These results suggest that some AMPs could exploit their intrinsic aggregation properties, by triggering bacteria agglutination as part of its mechanism of action as observed for a wealth source of AMPs in saliva, which provides a first barrier to bacteria adherence in the oral cavity [34]. Agglutinating activity has been reported crucial for the antimicrobial function of Eosinophil Cationic Protein (ECP) [35], a small cationic protein specifically secreted by eosinophil granules during inflammation processes with diverse antipathogen activities [36]–[38]. ECP displays high antimicrobial action, with a specific bacteria agglutination activity reported for Gram-negative bacteria, at a concentration range close to the minimal inhibitory concentration, a behavior that may represent an effective bactericidal mechanism *in vivo* [39].

In order to characterize the relation between AMPs, bacteria agglutination and amyloid aggregation, we have used ECP as a model of study. We present here a detailed characterization of protein-mediated bacteria agglutination and prove the contribution of an aggregation prone domain to the protein antimicrobial action. Complementary studies on model membranes provide a further understanding of the membrane damage process promoted by protein aggregation.

## Results/Discussion

ECP was previously reported to aggregate *in vivo* on both bacterial and eukaryotic cell surface without detectable internalization [39], [40]. Though these findings were essential to explain the antimicrobial and cytotoxic properties of ECP, the real nature of the aggregation process remained unknown. Besides, the protein has a high affinity towards lipopolysaccharides (LPS) [41] and agglutinates all tested Gram-negative strains [42]. On the other hand, ECP has been reported to form amyloid-like aggregates *in vitro* at specific conditions due to a hydrophobic patch located at the N-terminus. Remarkably, protein amyloid-like aggregation was efficiently abolished by mutating Ile 13 to Ala [28]. The screening of the protein primary structure [43]–[45] and the design of derived peptides [42], [46] also allocated the antimicrobial region at the N-terminus. As the antimicrobial and amyloid active segments of the protein colocalize [28], [35], [42], [46], it is tempting to hypothesize that bacteria agglutination by ECP could be directly dependent on an amyloid-like aggregation process. This hypothesis raises some exciting questions: (i) Is cell agglutination required for antimicrobial activity? (ii) Is cell agglutination mediated by protein aggregation at the bacteria surface? (iii) Are aggregates formed on the surface of bacteria of amyloid nature?

### Bacteria cell agglutination and antimicrobial activities

**Figure 1:**
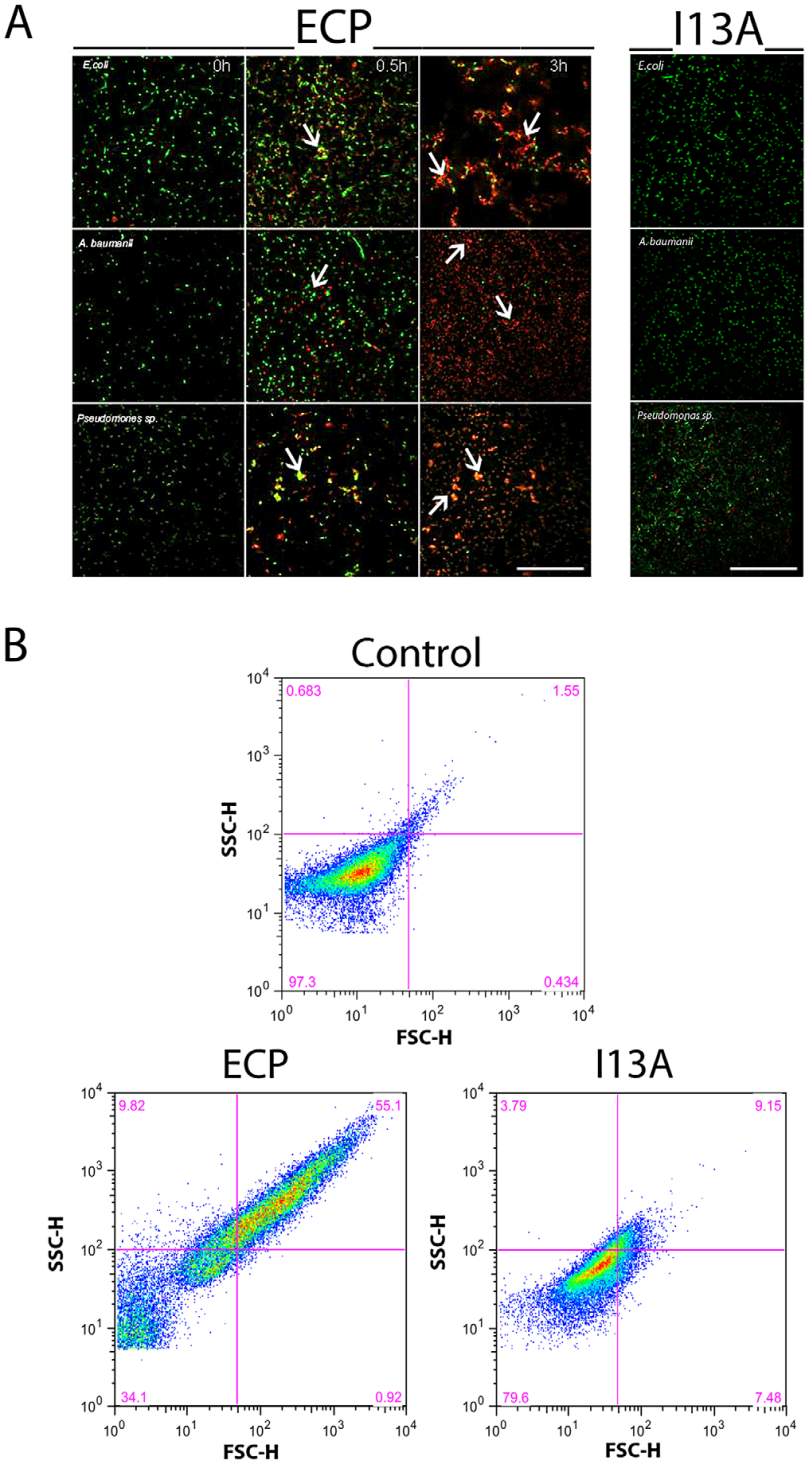
ECP but not I13A is able to agglutinate bacteria. (A) *E. coli*, *P. aeruginosa and A. baumanii* cells were incubated with 5 µM of ECP or I13A mutant in microscopy plates during 4 h and stained with syto9 (live cells, green) and propidium iodide (dead cells, red). Images were taken at 0, 0.5 and 3 h using a Leica SP2 confocal microscopy as described in the *Materials and Methods* section. Scale bar represents 50 µm. Arrows were depicted to show cell agglutination. Images depicted are representative from two independent experiments. (B) *E. coli* cells were incubated with 5 µM of ECP or I13A mutant during 4 h and samples were analyzed using a FACSCalibur cytometer. FSC-H is the low-angle forward scattering, which is roughly proportional to the diameter of the cell and SSC-H is the orthogonal or side scattering, which is proportional to cell granularity or complexity. Agglutination is registered as an increase in both scattering measures. In all experiments cell cultures were grown at exponential phase (OD_600_ = 0.2) and incubated with proteins in 10 mM sodium phosphate buffer, 100 mM NaCl, pH 7.4. The plots are representative of three independent experiments.

To address the first question we compared the antimicrobial action of wild type ECP (wtECP) with the I13A mutant, previously described to be unable to form aggregates *in vitro* [28]. The antimicrobial assays reveal that, while wtECP has an average minimal inhibitory concentration (MIC) value around 0.5–1 µM, the I13A mutant is unable to kill bacteria even at 5 µM concentration (Table 1). To further correlate ECP antimicrobial and agglutination activities we studied bacteria cell cultures by confocal microscopy using the SYTO9/Propidium iodide nucleic acid fluorescent labels that allow registering both cell agglutination and viability over time. Interestingly, wtECP can agglutinate Gram-negative bacteria before a viability decrease is observed (Figure 1A), however no cell agglutination takes place when bacteria are incubated with the I13A variant, even after 4 hours (Supporting Information Figure S1). These results are also supported by minimal agglutination concentrations (MAC) close to the MIC values (Table 1) and by FACS experiments showing that wtECP but not I13A mutant is able to agglutinate *E. coli* cells (Figure 1B). Thus, ECP antimicrobial activity on Gram-negative strains is strongly affected when abolishing the agglutination behavior (Ile13 to Ala mutation).

**Table 1:** Antimicrobial (MIC_100_) and agglutinating (MAC) activities of wtECP and I13A mutant in Gram-negative strains.

	MIC_100_ (µM)		MIC_100_ (µM)		MAC (µM)		MAC (µM)	
	Phosphate buffer^a^		MH medium^b^		Phosphate buffer^a^		MH medium^b^	
	ECP	I13A	ECP	I13A	ECP	I13A	ECP	I13A
***E. coli***	0.40±0.10	>5	0.45±0.10	>5	0.25±0.1	>5	0.25±0.1	>5
***P. aeruginosa***	0.60±0.15	>5	0.90±0.20	>5	0.5±0.1	>5	0.5±0.1	>5
***A. baumanii***	0.75±0.15	>5	1.25±0.20	>5	1.0±0.2	>5	1.0±0.2	>5

a Bacteria were grown in LB medium and incubated with proteins in 10 mM NaH_2_PO_4_, 100 mM NaCl pH 7.4.

b Bacteria were grown and incubated with proteins in Mueller–Hinton II broth.

### Protein aggregation on membrane models

**Figure 2:**
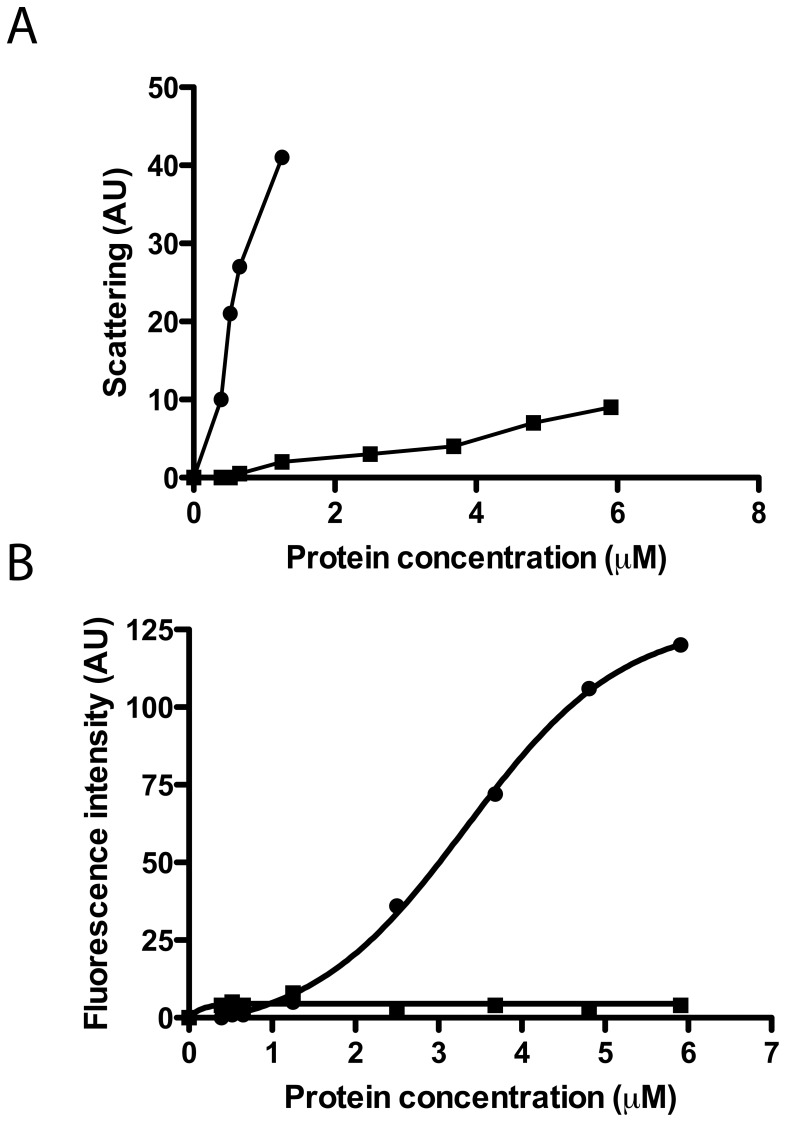
Liposome agglutination and leakage activity. wtECP (circles) and I13A mutant (squares) were incubated with liposomes and the agglutination (A) and leakage (B) were followed at increasing protein concentrations (1–6 µM). Agglutination was measured as light scattering (470 nm) at 90° from the beam source in a 10 mM Tris-HCl, 100 mM NaCl, pH 7.4 buffer and leakage was followed using the ANTS/DPX assay in the same buffer as described in the *Materials and Methods* section.

To further analyze the protein agglutination mechanism, we tested the wtECP and I13A mutant action on a simpler biophysical system such as phospholipid membranes where liposome agglutination is registered as a function of protein concentration. In contrast to wtECP, I13A mutant completely looses the ability to agglutinate membranes (Figure 2A). In particular, when following wtECP agglutinating activity as a function of ionic strength, we observe that liposome agglutination is enhanced at high NaCl concentration (Supporting Information, Figure S2). These results suggest that vesicle agglutination is promoted by hydrophobic interactions. Even more, leakage activity in model membranes is also lost for I13A mutant (Figure 2B), meaning that protein aggregation on the membrane surface is important not only for agglutination but also for later membrane permeabilization. These results are entirely consistent with those described above for bacteria cell cultures where the Ile to Ala mutation not only abolishes the cell agglutinating activity of ECP but also its bactericidal action.

### Agglutination mediated by protein aggregation

**Figure 3:**
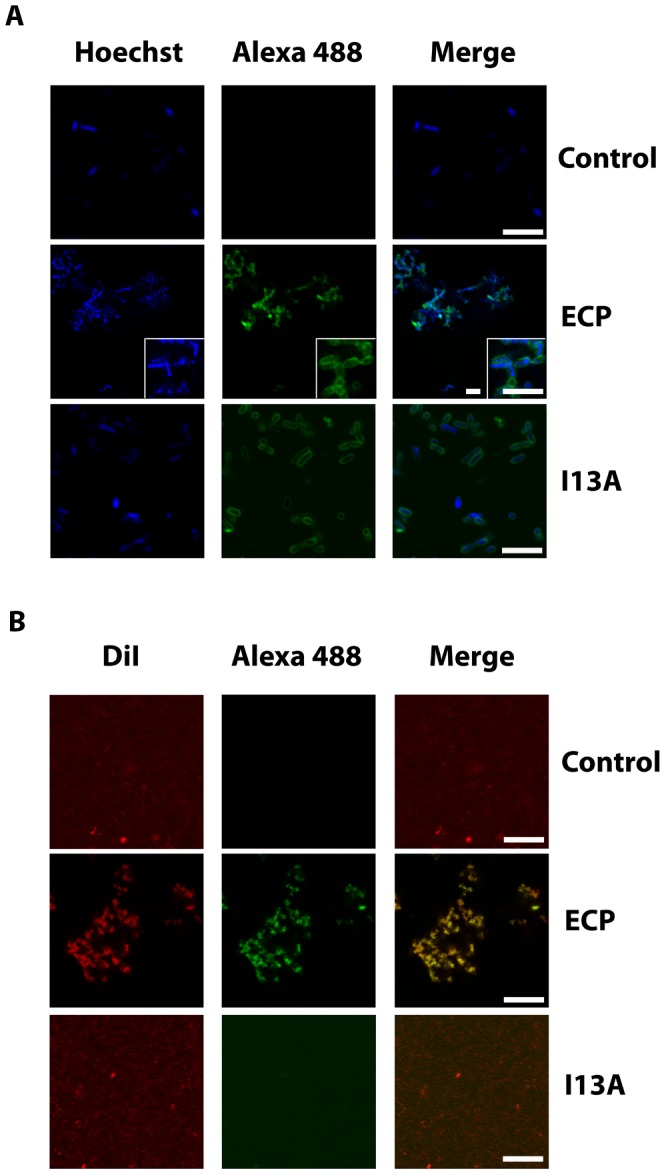
ECP and I13A mutant bind to the surface of bacteria and membranes. (A) *E. coli* bacteria cells stained with Hoechst (blue signal) were incubated with 5 µM of either wtECP or I13A mutant (both labeled with Alexa Fluor 488; green signal) for 4 h and visualized by confocal microscopy. In all experiments cell cultures were grown at exponential phase (OD_600_ = 0.2) and incubated with proteins in 10 mM sodium phosphate buffer, 100 mM NaCl, pH 7.4. (B) 500 µl of 200 µM LUV liposomes stained with DiI (red signal) were incubated with 5 µM of either wtECP or I13A mutant (both labeled with Alexa Fluor 488; green signal) for 4 h and visualized using a Leica SP2 confocal microscope. Scale bars are 5 µm length in all images and insight captions. Images depicted are representative from two independent experiments.

Next, to address the question whether cell agglutination is consistently driven by protein aggregation at the bacteria surface, we incubated bacteria cultures with ECP and visualized the samples using confocal microscopy. Our results show that wtECP binds to the bacteria surface and a strong protein signal is registered at the aggregation zones (Figure 3A). On the contrary, though cell interaction is maintained for the I13A mutant, agglutination is observed neither in bacteria cell cultures nor in model membranes (Figures 3A and 3B). As expected, for model membranes we show that only wtECP is able to promote agglutination (Figure 3B). Therefore, we conclude that protein aggregation on the cellular surface is required for bacteria agglutination, which turns to be essential for the antimicrobial action. Agglutination is also observed in the presence of 20% plasma in a similar extent, suggesting that ECP agglutination is likely to take place in the physiological context (Supplementary Information Figure S3). As previously mentioned, ECP binding to bacteria is favored by interactions with the LPS outer membrane [35], [41], [47]. Consistently, we show here that LPS binding activity is lost for the I13A mutant, when compared with wtECP (Supplementary Information Figure S4).

### In situ follow-up of amyloid aggregates

**Figure 4:**
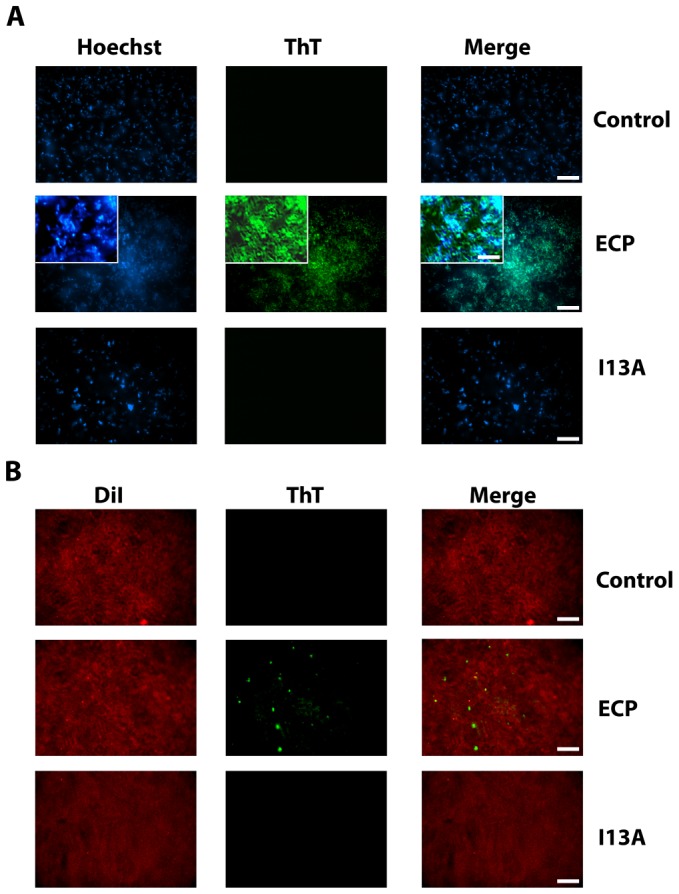
ECP but not I13A form amyloid-like aggregates on the surface of bacteria and membranes. (A) *E. coli* bacteria cells stained with Hoechst (blue signal) were incubated with unlabeled wtECP or I13A mutant for 4 h, stained with ThT (green signal) and visualized by TIRF microscopy. In all experiments cell cultures were grown at exponential phase (OD_600_ = 0.2) and incubated with proteins in 10 mM sodium phosphate buffer, 100 mM NaCl, pH 7.4. (B) Planar lipid bilayers prepared as described in the *Materials and Methods* section (stained with DiI; red signal) were incubated with 5 µM of either unlabeled wtECP or I13A mutant for 4 h, stained with 25 µM ThT (green signal) and visualized using a Olympus FluoView 1000 TIRF microscope. Scale bar represents 20 µm (5 µm in the insight caption). Images depicted are representative from two independent experiments.

At this point however, the nature of the protein aggregates remained unknown. Thus, having previously shown that ECP is able to form amyloid-like aggregates *in vitro*, we decided to test if the observed aggregates have an amyloid-like structure using the amyloid-diagnostic dyes Thioflavin-T and Congo Red. When bacteria cultures are incubated with non-labeled wtECP, stained with ThT and visualized by total internal reflection fluorescence (TIRF) microscopy, we show that wtECP amyloid-like aggregates are located also at the cell surface (Figure 4A) similarly as what we observe for Alexa labeled wtECP (Figure 3A). Consistently, no staining is observed for non-incubated cultures and for the I13A mutant (Figure 4A). Moreover, upon bacteria incubation with wtECP, a red shift in the Congo Red spectrum is observed (Supplementary Information Figure S5A), revealing that the protein amyloid-like aggregation is triggered upon incubation with bacteria cultures.

Though ECP was previously shown to form amyloid-like aggregates *in vitro* only at low pH after a long incubation time (1–2 weeks), amyloid-like structures observed here are detected after only 4 hours of incubation. However, it is well known that some proteins can accelerate its aggregation kinetics in the presence of membrane-like environments [48]–[50]. Our results show that wtECP is able to form fibrillar-like aggregates on model membranes with an average size of 845±150 nm (Figure 4B), comparable in size with the wtECP aggregates observed *in vitro* in the absence of lipid membranes (∼150 nm) [28]. In fact, when tested for ThT binding, we observe aggregates with similar size (Figure 4B). When wtECP is incubated with model membranes and tested for Congo Red binding, we obtain again a noticeable spectral shift (Supplementary Information Figure S5B). To complete these results we have also performed all the experiments detailed above using the I13A mutant and found it to be unable to form amyloid-like aggregates (Figure 4).

### Conclusions

The results presented here for ECP reinforce the hypothesis that an amyloid-like aggregation process is taking place in the bacteria surface that drives bacteria cell agglutination, which is essential for the antimicrobial activity of the protein. In summary, after binding to the bacteria surface, a rearrangement of the protein could take place, exposing the hydrophobic N-terminal patch of the protein. Following, the aggregation process would start promoting the agglutination of the bacteria cells through the aggregation of the surface-attached protein molecules. The formation of aggregates on the bacteria surface will disrupt the lipopolysaccharide bilayer of Gram-negative cells exposing the internal cytoplasmatic membrane to the protein action, promoting the membrane disruption and eventually the bacteria killing.

Cell agglutinating activity provides a particularly appealing feature that may contribute to the clearance of bacteria at the infectious focus. In this sense, bacteria agglutination would prepare the field before host phagocytic cells enter in the scene [33]. However, despite the interest in the pharmaceutical industry to identify the structural determinants for bacteria cell agglutination, bibliography on that subject is scarce and only few agglutinating antimicrobial proteins are described in the literature. Excitingly, there may be other proteins and peptides with similar characteristics that also follow the proposed model. Hence, the agglutinating mechanism may represent a more generalized process that may derivate in amyloid deposit formation at bacterial infection focuses.

Besides, it has been reported that systematic exposure to inflammation may represent a risk factor on developing Alzheimer's disease [51], [52] and other types of dementia [53]. Some studies have also demonstrated that the release of inflammatory mediators can also cause generalized cytotoxicity. In particular, ECP has been discovered to be cytotoxic [40], [54] and neurotoxic, causing the Gordon phenomenon after injection intratechally in rabbits [55]. Therefore, our results suggest that the release of inflammatory mediators after infection (like AMPs) may either seed the aggregation processes in the brain and/or influence the membrane biophysical properties to trigger neurotoxicity and aggregation events.

## Materials and Methods

### MIC (Minimal Inhibitory Concentration) and MAC (Minimal Agglutination Concentration) determination

Antimicrobial activity was expressed as the MIC_100_, defined as the lowest protein concentration that completely inhibits microbial growth. MIC of each protein was determined from two independent experiments performed in triplicate for each concentration. Bacteria were incubated at 37°C overnight in Mueller-Hinton II (MHII) broth and diluted to give approximately 5·10^5^ CFU/mL. Bacterial suspension was incubated with proteins at various concentrations (0.1–5 µM) at 37°C for 4 h either in MHII or 10 mM sodium phosphate buffer, 100 mM NaCl, pH 7.4. Samples were plated onto Petri dishes and incubated at 37°C overnight.

For MAC determination, bacteria cells were grown at 37°C to mid-exponential phase (OD_600_ = 0.6), centrifuged at 5000×g for 2 min, and resuspended in 10 mM sodium phosphate buffer, 100 mM NaCl, pH 7.4, in order to give an absorbance of 0.2 at 600 nm. A 200 µL aliquot of the bacterial suspension was incubated with proteins at various (0.1–10 µM) concentrations at 25°C for 4 h. Aggregation behavior was observed by visual inspection and minimal agglutinating concentration expressed as previously described [42].

### Fluorescence-Assisted Cell Sorting (FACS) assay

Bacteria cells were grown at 37°C to mid-exponential phase (OD_600_ = 0.6), centrifuged at 5000×g for 2 min, resuspended in 10 mM sodium phosphate buffer, 100 mM NaCl, pH 7.4 or the same buffer supplemented with 20% plasma to give a final OD_600_ = 0.2 and preincubated for 20 min. A 500 µL aliquot of the bacterial suspension was incubated with 5 µM of wtECP or I13A mutant during 4 h. After incubation, 25000 cells were subjected to FACS analysis using a FACSCalibur cytometer (BD Biosciences, New Jersey) and a dot-plot was generated by representing the low-angle forward scattering (FSC-H) in the x-axis and the side scattering (SSC-H) in the y-axis to analyze the size and complexity of the cell cultures. Results were analyzed using FlowJo (Tree Star, Ashland, OR).

### Bacteria viability assay

Bacteria viability assays were performed as described before [39]. Briefly, bacteria were incubated in 10 mM sodium phosphate buffer, 100 mM NaCl, pH 7.4 with 5 µM of wtECP or I13A mutant and then stained using a syto 9/propidium iodide 1∶1 mixture. The viability kinetics were monitored using a Cary Eclipse Spectrofluorimeter (Varian Inc., Palo Alto, CA, USA). To calculate bacterial viability, the signal in the range 510–540 nm was integrated to obtain the syto 9 signal (live bacteria) and from 620–650 nm to obtain the propidium iodide signal (dead bacteria). Then, the percentage of live bacteria was represented as a function of time.

### Liposome agglutination and leakage assay

 The ANTS/DPX liposome leakage fluorescence assay was performed as previously described [56]. Briefly, a unique population of LUVs of DOPC/DOPG (3∶2 molar ratio) lipids was obtained containing 12.5 mM ANTS, 45 mM DPX, 20 mM NaCl, and 10 mM Tris/HCl, pH 7.4. The ANTS/DPX liposome suspension was diluted to 30 µM concentration and incubated at 25°C in the presence of wtECP or I13A mutant. Leakage activity was followed by monitoring the increase of the fluorescence at 535 nm.

For liposome agglutination, 200 µM LUV liposomes were incubated in 10 mM phosphate buffer, pH 7.4, containing 5 to 100 mM NaCl, in the presence of 5 µM wtECP or I13A mutant and the scattering signal at 470 nm was collected at 90° from the beam source using a Cary Eclipse Spectrofluorimeter (Varian Inc., Palo Alto, CA, USA) [57].

### Confocal microscopy

 Experiments were carried out in 35 cm^2^ plates with a glass coverslip. For phospholipid membranes, 500 µl of 200 µM LUV liposomes (prepared as described in Supplementary Information) were incubated with 5 µM wtECP or I13A mutant for 4 h in 10 mM sodium phosphate buffer, 100 mM NaCl, pH 7.4. For bacteria, 500 µl of *E. coli* cells (OD_600_ = 0.2) were incubated with 5 µM wtECP or I13A mutant for 4 h in 10 mM sodium phosphate buffer, 100 mM NaCl, pH 7.4. RNase A was used always as a negative control. Samples of both liposomes and bacteria were imaged using a laser scanning confocal microscope (Olympus FluoView 1000 equipped with a UPlansApo 60× objective in 1.4 oil immersion objective, United Kingdom). wtECP and I13A mutant labeled with Alexa Fluor 488 were excited using a 488-nm argon laser (515–540 nm emission collected) and Vibrant DiI was excited using an orange diode (588–715 nm emission collected).

### TIRF microscopy

To study the interaction of proteins with lipid membranes, planar supported lipid bilayers were used (Supplementary Information). When using bacteria, glass coverslips were previously treated with 0.1% poly-L-lysine to ensure that samples will adhere to the surface. 500 µl of *E. coli* cells (OD_600_ = 0.2) were incubated with 5 µM wtECP or I13A mutant for 4 h and then transferred to poly-L-lysine treated microscopy plates and incubated for 15 minutes. To remove unattached cells, plates were washed twice with 10 mM sodium phosphate, 100 mM NaCl, pH 7.4 buffer. RNase A was used always as a negative control. Images were captured using a laser scanning confocal microscope (Olympus FluoView 1000 equipped with a PlansApo 60× TIRF objective in 1.4 oil immersion objective, United Kingdom) using the same conditions as described for confocal microscopy experiments. Thioflavin T (ThT) was used to detect amyloid aggregates. In this case, samples were incubated for 4 h with unlabeled proteins as described before and then incubated with ThT at 25 µM final concentration for 15 minutes. Then, plates were washed twice with 10 mM sodium phosphate, 100 mM NaCl buffer, pH 7.4 to remove unattached cells and ThT excess.

## Supporting Information

Figure S1 **Bacteria agglutination mediated by wtECP and the I13A mutant.** *E. coli* bacteria cells were grown at exponential phase (OD_600_ = 0.2) and incubated with 0.5 µM wtECP (A) or I13A (B) in 10 mM phosphate buffer, 100 mM NaCl, pH 7.5 for 4 h. Images were taken using a Leica magnificator. wtECP incubated bacteria samples were also observed under 40× (C) and 100× (D) magnification to reveal more details on bacteria aggregates. Images were taken using a Leica optical microscope.
(TIF)

Figure S2 **Liposome agglutination mediated by wtECP and I13A mutant at increasing ionic strength.** Liposomes prepared as described in the *Materials and Methods* section were incubated with increasing concentrations of wtECP (circles) or I13A mutant (squares) at 5 mM (A), 50 mM (B) and 100 mM (C) NaCl in a 10 mM phosphate buffer, pH 7.5. The formation of liposome aggregates was followed as an increase in the light scattering signal at 90° from the beam.
(TIF)

Figure S3 **ECP is able to agglutinate bacteria cells in plasma.** *E. coli* cells were incubated with 5 µM of ECP during 4 h and samples were analyzed using a FACSCalibur cytometer. FSC-H is the low-angle forward scattering, which is roughly proportional to the diameter of the cell and SSC-H is the orthogonal or side scattering, which is proportional to cell granularity or complexity. Agglutination is registered as an increase in both scattering measures. In all experiments, cell cultures were grown at exponential phase (OD_600_ = 0.2) and incubated with proteins in 20% plasma diluted in 10 mM sodium phosphate buffer, 100 mM NaCl, pH 7.5. The plots are representative of three independent experiments.
(TIF)

Figure S4 **wtECP and I13A mutant binding to bacteria LPS.** LPS were incubated with increasing concentrations of wtECP (circles) or I13A mutant (squares) in a 10 mM phosphate buffer, 100 mM NaCl, pH 7.5. Binding to bacteria LPS was registered as a fluorescence increase of the BODIPY-cadaverine reporter as described in the *Materials and Methods* section. The occupancy factor denotes the decrease of the LPS-bound dye fraction after protein addition.
(TIF)

Figure S5 **Protein aggregates bind to Congo Red dye.** (A) *E. coli* (circles) and *P. aeruginosa* (triangles) bacteria cells were incubated 4 h with wtECP and assayed for Congo Red binding as described in the *Materials and Methods* section. (B) Liposomes at 10 µM (black circles), 200 µM (grey circles) and 1 mM (white squares) lipid concentration were incubated with wtECP and assayed for Congo Red binding as described in the *Materials and Methods* section. Congo Red differential spectra were obtained by subtracting both the signal corresponding to the protein and the lipid/bacteria in the presence of the dye. The vertical line at 480 nm represents the spectrum of Congo Red alone. Incubation of I13A mutant with both bacteria and membranes did not display any significant spectral shift.
(TIF)

Protocol S1 This file contains additional details for the Materials and Methods section.
(DOCX)
