# Supplementary figures and images for: Exploring New Biological Functions of Amyloids: Bacteria Cell Agglutination Mediated by Host Protein Aggregation
Source: PLoS Pathog. 2012 Nov 1;8(11):e1003005. doi: 10.1371/journal.ppat.1003005 (PMC3486885; doi:10.1371/journal.ppat.1003005)

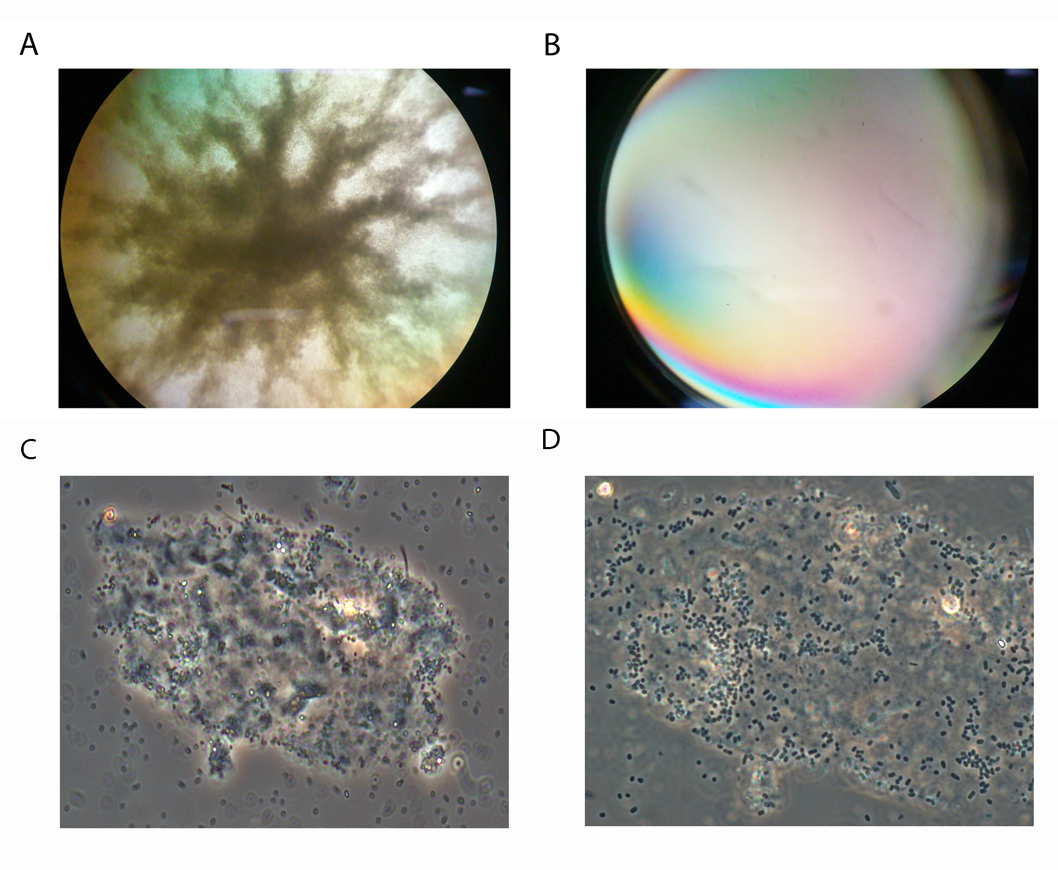

Supplement: Figure S1 — Bacteria agglutination mediated by wtECP and the I13A mutant. E. coli bacteria cells were grown at exponential phase (OD600 = 0.2) and incubated with 0.5 µM wtECP (A) or I13A (B) in 10 mM phosphate buffer, 100 mM NaCl, pH 7.5 for 4 h. Images were taken using a Leica magnificator. wtECP incubated bacteria samples were also observed under 40× (C) and 100× (D) magnification to reveal more details on bacteria aggregates. Images were taken using a Leica optical microscope. (TIF) [file ppat.1003005.s001.tif]

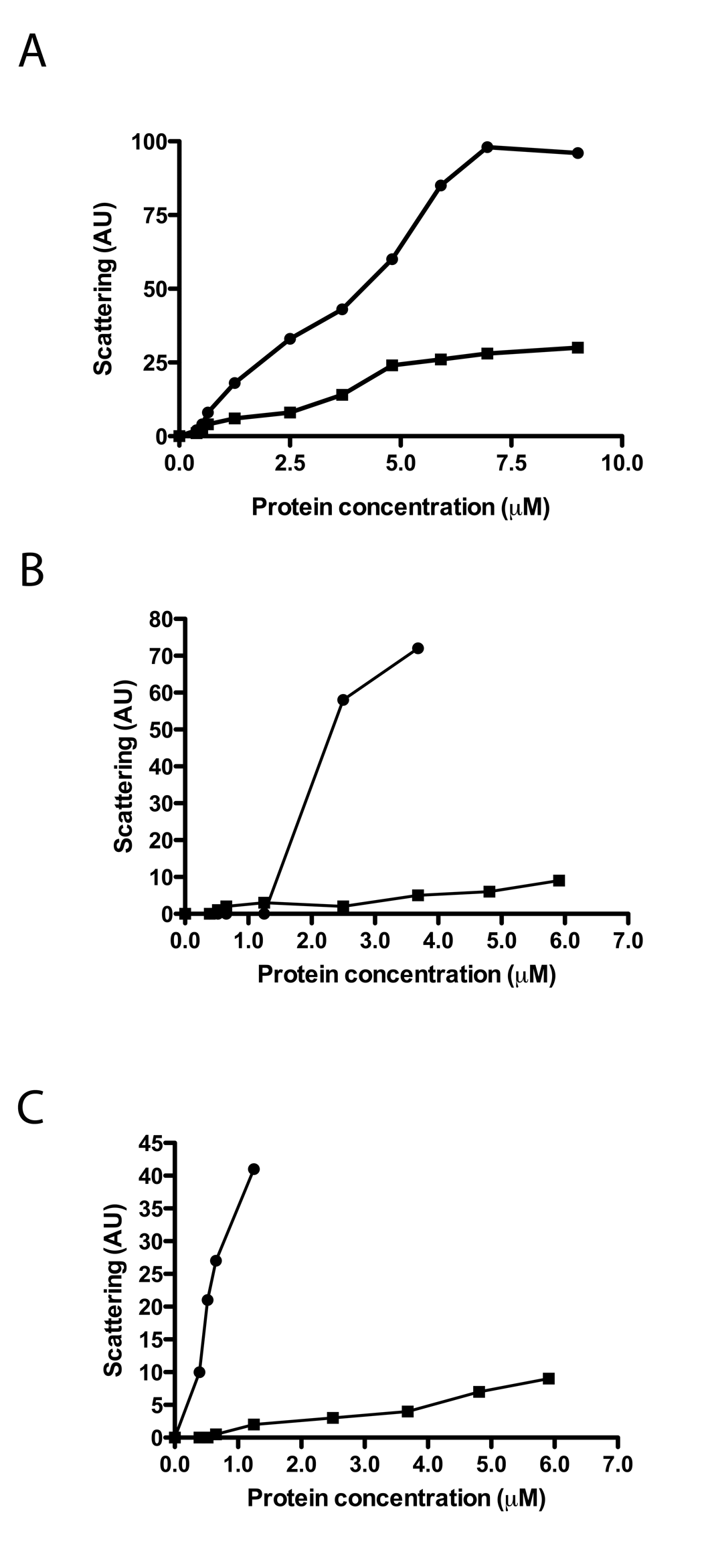

Supplement: Figure S2 — Liposome agglutination mediated by wtECP and I13A mutant at increasing ionic strength. Liposomes prepared as described in the Materials and Methods section were incubated with increasing concentrations of wtECP (circles) or I13A mutant (squares) at 5 mM (A), 50 mM (B) and 100 mM (C) NaCl in a 10 mM phosphate buffer, pH 7.5. The formation of liposome aggregates was followed as an increase in the light scattering signal at 90° from the beam. (TIF) [file ppat.1003005.s002.tif]

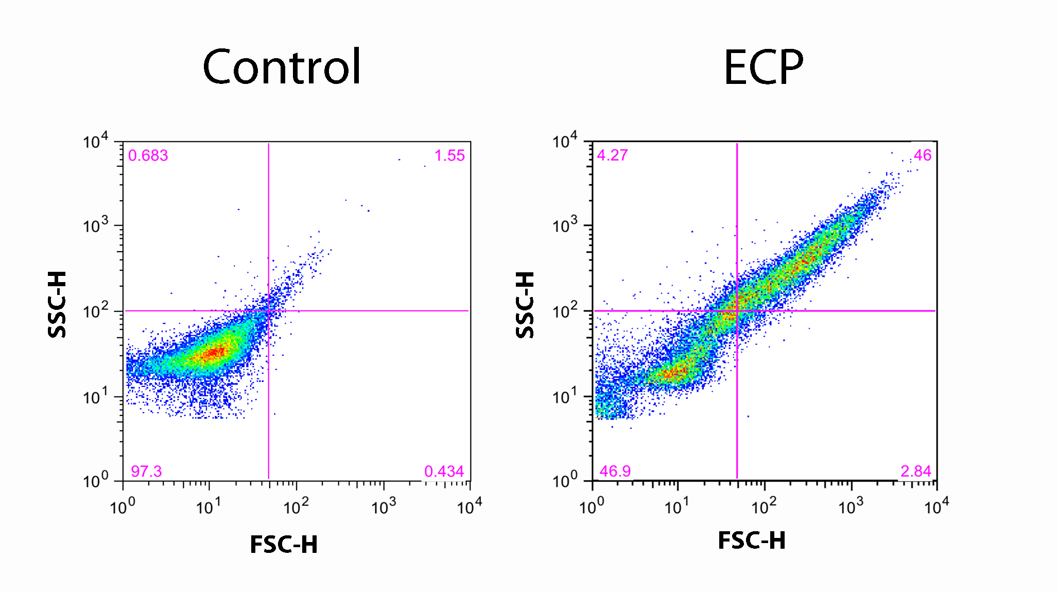

Supplement: Figure S3 — ECP is able to agglutinate bacteria cells in plasma. E. coli cells were incubated with 5 µM of ECP during 4 h and samples were analyzed using a FACSCalibur cytometer. FSC-H is the low-angle forward scattering, which is roughly proportional to the diameter of the cell and SSC-H is the orthogonal or side scattering, which is proportional to cell granularity or complexity. Agglutination is registered as an increase in both scattering measures. In all experiments, cell cultures were grown at exponential phase (OD600 = 0.2) and incubated with proteins in 20% plasma diluted in 10 mM sodium phosphate buffer, 100 mM NaCl, pH 7.5. The plots are representative of three independent experiments. (TIF) [file ppat.1003005.s003.tif]

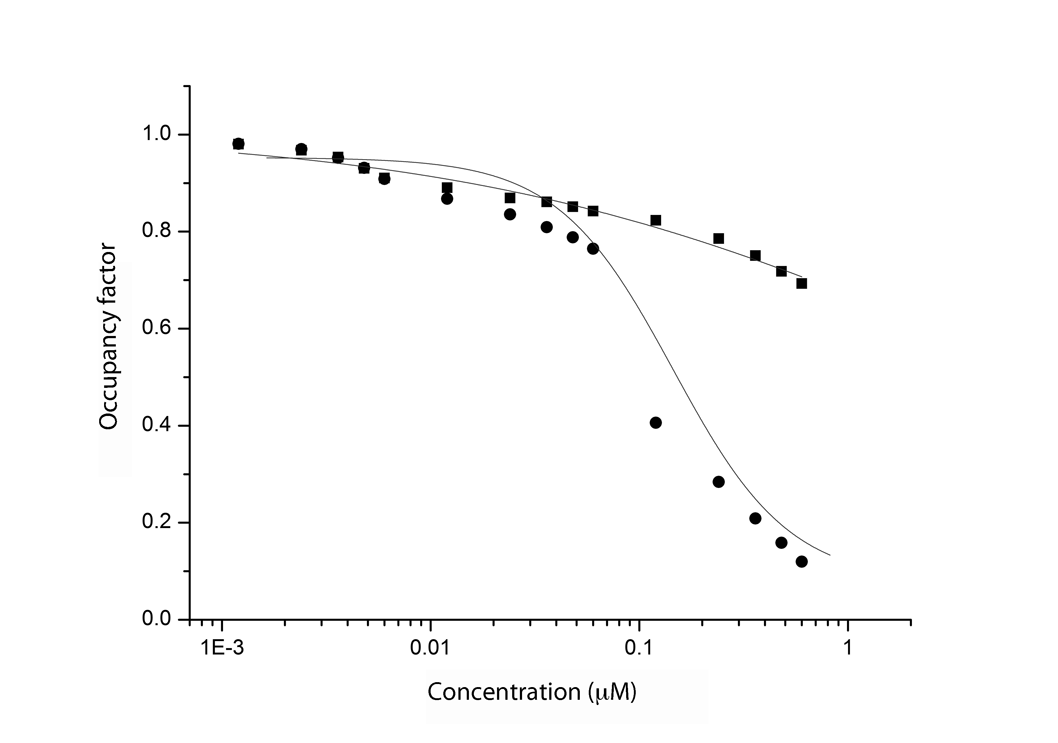

Supplement: Figure S4 — wtECP and I13A mutant binding to bacteria LPS. LPS were incubated with increasing concentrations of wtECP (circles) or I13A mutant (squares) in a 10 mM phosphate buffer, 100 mM NaCl, pH 7.5. Binding to bacteria LPS was registered as a fluorescence increase of the BODIPY-cadaverine reporter as described in the Materials and Methods section. The occupancy factor denotes the decrease of the LPS-bound dye fraction after protein addition. (TIF) [file ppat.1003005.s004.tif]

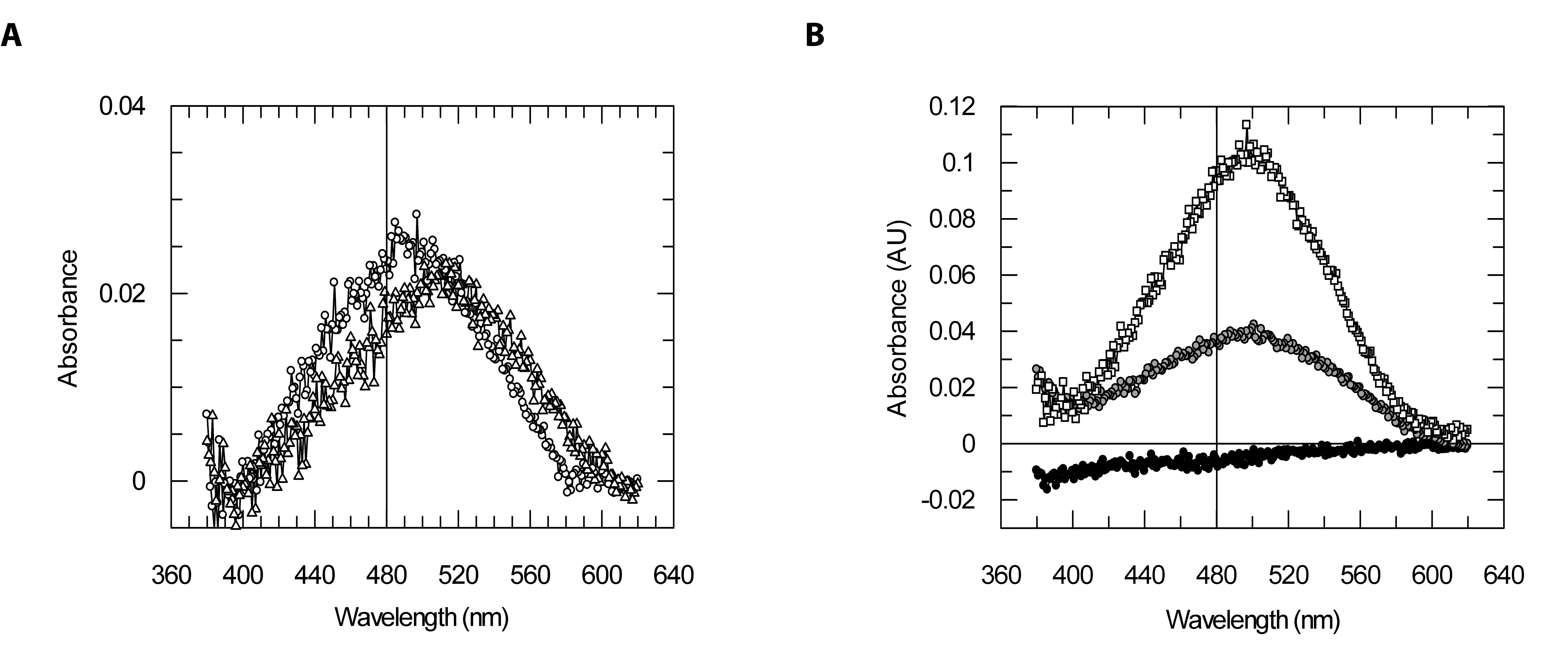

Supplement: Figure S5 — Protein aggregates bind to Congo Red dye. (A) E. coli (circles) and P. aeruginosa (triangles) bacteria cells were incubated 4 h with wtECP and assayed for Congo Red binding as described in the Materials and Methods section. (B) Liposomes at 10 µM (black circles), 200 µM (grey circles) and 1 mM (white squares) lipid concentration were incubated with wtECP and assayed for Congo Red binding as described in the Materials and Methods section. Congo Red differential spectra were obtained by subtracting both the signal corresponding to the protein and the lipid/bacteria in the presence of the dye. The vertical line at 480 nm represents the spectrum of Congo Red alone. Incubation of I13A mutant with both bacteria and membranes did not display any significant spectral shift. (TIF) [file ppat.1003005.s005.tif]
