# Supplementary material for: Exploring New Biological Functions of Amyloids: Bacteria Cell Agglutination Mediated by Host Protein Aggregation
Source: PLoS Pathog. 2012 Nov 1;8(11):e1003005. doi: 10.1371/journal.ppat.1003005 (PMC3486885; doi:10.1371/journal.ppat.1003005)
Supplement: Protocol S1 — This file contains additional details for the Materials and Methods section. (DOCX) [file ppat.1003005.s006.docx]

**Materials and Methods**

*Materials and Strains*

1,2-Dioleoyl-sn-glycero-3-phosphocholine (DOPC), 1,2-dioleoyl-sn-glycero-3-[phospho-rac-(1-glycerol)] (DOPG), Dipalmitoil-sn-glycero-3-phosphocholine (DPPC) and Dimiristoil-sn-glycero-3--[phospho-rac-(1-glycerol)] (DMPG) were from Avanti Polar Lipids, Birmingham, AL. LPS from *E. coli* was purchased from Sigma Aldrich.

8-Aminonaphthalene-1,3,6-trisulfonic acid disodium salt (ANTS), p-xylene-bispyridiniumbromide (DPX), BC (BODIPY TR cadaverine, where BODIPY is boron dipyrromethane (4,4-difluoro- 4-bora-3a,4a-diaza-s-indacene) were purchased from Invitrogen (Carlsbad, CA). Alexa Fluor 488 Protein Labelling kit and Vibrant DiI cell-labeling solution were from Molecular Probes (Invitrogen, Carlsbad, CA). pET11 expression vector and *E. coli* BL21(DE3) cells were from Novagen, Madison,WI.

Strains used were *Escherichia coli* (BL21, Novagen), *Acinetobacter baumannii* (ATCC 15308) and *Pseudomonas sp* (ATCC 15915). Human plasma was extracted from a pool of healthy donor’s blood.

*Preparation of recombinant proteins*

I13A ECP mutation was incorporated using the Quick-change mutagenesis kit from Invitrogen (Carslbad, CA). Recombinant ECP and I13A mutant were obtained as previously described ([1](#_ENREF_1), [2](#_ENREF_2)). I13A ECP mutant was sequenced before expression and protein identity was checked by MALDI-TOF mass spectrometry.

*Protein fluorescent labeling*

ECP and I13A mutant were labeled with the Alexa Fluor 488 fluorophor, following the manufacturer's instructions, as previously described ([3](#_ENREF_3)). To 0.5 mL of a 2 mg/mL protein solution in phosphate saline buffer (PBS), 50 μL of 1 M sodium bicarbonate, pH 8.3, were added. The protein is incubated for 1 h at room temperature, with the reactive dye, with stirring, following the manufacturer's conditions. The labeled protein was separated from the free dye by a PD10-desalting column.

*LUV liposome preparation*

Large unilamellar vesicles (LUVs) containing DOPC/DOPG (3:2 molar ratio) of a defined size (about 100 nm) were prepared as previously described ([4](#_ENREF_4)). LUVs were obtained from a vacuum-drying lipid chloroform solution by extrusion through 100 nm polycarbonate membranes. The lipid suspension was frozen and thawed ten times prior to extrusion. A 1 mM stock solution of liposome suspension in 10 mM phosphate buffer, 100 mM NaCl, pH 7.4 was prepared.

*Formation of supported lipid bilayers (SLBs).*

Supported lipid bilayers were prepared as described ([5](#_ENREF_5)). Briefly, we dissolved DMPG and DPPC in CHCl_3_/methanol 2/1(v/v) to obtain a 5 mM stock solution of each lipid. 80 μl of DMPG stock solution and 120 μl of DPPC stock solution were mixed in a glass tube to obtain a 3:2 DPPC/DOPG mixture. Then the lipid mixture was evaporated under nitrogen and dried in a desiccator under vacuum for 2 h. The dried lipid ﬁlm was resuspended in 1 ml of a 10 mM Tris, 100 mM NaCl, 3 mM CaCl_2_ buffer adjusted at pH 7.4 with HCl to give a 1 mM ﬁnal lipid concentration of multilamellar large vesicles (MLVs) suspension. In order to obtain small unilamelar vesicles (SUVs), the lipid suspension was sonicated to clarity (ﬁve cycles of 2 min) using a water bath sonicator at 60ºC. Then, 500 μl of the SUV suspension was placed in contact with the glass coverslip of a 35 cm^2^ microscopy plate and heated for 45 min at 60 ºC. After slowly cooling down the system to room temperature, the samples were rinsed carefully with a 10 mM phosphate buffer, 100 mM NaCl buffer adjusted at pH 7.4 to remove the SUV excess.

*LPS Binding Assay*

LPS binding was assessed using the fluorescent probe Bodipy TR cadaverine (BC) as described ([6](#_ENREF_6)). Briefly, the displacement assay was performed by the addition of 1-2 μL aliquots of wtECP or I13A mutant solution to 1 mL of a continuously stirred mixture of LPS (10 μg/mL) and BC (10 μM) in 10 mM phosphate buffer, 100 mM NaCl, pH 7.5. Fluorescence measurements were performed on a Cary Eclipse spectrofluorimeter. The BC excitation wavelength was 580 nm, and the emission wavelength was 620 nm. The excitation slit was set at 2.5 nm, and the emission slit was set at 20 nm. Final values correspond to an average of four replicates and were the mean of a 0.3 s continuous measurement. Occupancy factor was calculated as described previously ([6](#_ENREF_6)).

*Binding to Congo Red*

Binding of ECP to Congo red was tested using a Varian CARY- 100 spectrophotometer (Palo Alto, CA) to perform the spectroscopic band-shift assay. A total of 5 μL of 10 mg/mL protein solution was diluted in 45 μL of 10 mM sodium phosphate, 100 mM NaCl, and pH 7.5 buffer containing 15 μM Congo red for recording the spectra of the protein alone. For recording the shift after binding to liposomes, increasing concentration of liposomes, prepared as described previously, were added to the buffer. For recording the shift after binding to bacteria, a sufficient amount of cells was added to the buffer to reach an OD_600_ = 0.2. Samples were maintained 5 min at 25 °C before analysis. Congo Red differential spectra were obtained by subtracting both the signal corresponding to the protein and the lipid/bacteria in the presence of the dye. Samples incubated with the I13A mutant or lysed bacteria do not show a significant increase of Congo Red spectral shift.

**REFERENCES**

1. Boix E*, et al.* (1999) Kinetic and product distribution analysis of human eosinophil cationic protein indicates a subsite arrangement that favors exonuclease-type activity. *J Biol Chem* 274(22):15605-15614.

2. Torrent M, Odorizzi F, Nogues MV, & Boix E (2010) Eosinophil cationic protein aggregation: identification of an N-terminus amyloid prone region. *Biomacromolecules* 11(8):1983-1990.

3. Torrent M*, et al.* (2009) Comparison of the membrane interaction mechanism of two antimicrobial RNases: RNase 3/ECP and RNase 7. *Biochim Biophys Acta* 1788(5):1116-1125.

4. Torrent M*, et al.* (2007) Topography studies on the membrane interaction mechanism of the eosinophil cationic protein. *Biochemistry* 46(3):720-733.

5. Mingeot-Leclercq MP, Deleu M, Brasseur R, & Dufrene YF (2008) Atomic force microscopy of supported lipid bilayers. *Nature protocols* 3(10):1654-1659.

6. Torrent M, Navarro S, Moussaoui M, Nogues MV, & Boix E (2008) Eosinophil cationic protein high-affinity binding to bacteria-wall lipopolysaccharides and peptidoglycans. *Biochemistry* 47(11):3544-3555.
